# "The Hospital" Nursing Mirror

**Published:** 1900-11-03

**Authors:** 


### "The Hospital" Nursing Mirror.

Being the Nursing Section of "The Hospital."

[Contributions for this Section of "The Hospital" should be addressed to the Editor, "The Hospital" Nursing Mirror, 28 & 29 Southampton Street, Strand, London, W.C.]

### Notes on News from the Nursing World.

### PRINCESS CHRISTIAN'S LOSS.

Nurses everywhere will learn with great regret of the sad event which converted Monday from a day of rejoicing into a day of mourning for the Queen and the Royal Family, while members of the Royal British Nurses' Association in particular will deeply sympathise with their illustrious President in the irreparable loss she has sustained by the death of her elder son, Prince Christian Victor. The news was quite unexpected, for Princess Christian was about to leave Cumberland Lodge to join in London's welcome to the City Volunteers when the telegram from Lord Roberts arrived. It was known that the gallant Prince was suffering from enteric fever, but no one seems to have feared that he would not recover. Princess Christian had only just commenced the resumption of her engagements after her autumn holiday; but it will now, of course, be some time before she is able to take an active interest in public matters. The absence of Prince Christian in Germany and of two of her children must have accentuated the force of the blow; but we are glad to hear that Her Royal Highness is bearing up bravely.

### STATE REGISTRATION OF NURSES.

The chief subject of interest to nurses discussed at the Brighton Conference last week was the work of women on Hospital Boards. Miss Louisa Stevenson, Governor of Edinburgh Infirmary, who read the first paper, did not, however, content herself with urging the claims of her sex to places on the governing bodies of hospitals. She proceeded to argue at length in favour of a comprehensive system of State registration of nurses, which she contended would promote the interests of the State, the general public, the hospitals, private patients, and nurses themselves. We do not think so, any more than we think that the interests of patients or nurses would gain by the municipalisation of hospitals; and it is a matter for regret that a policy open to so many objections should have been ventilated in the treatment of a question which really has no connection with it.

### SCARCITY OF NURSES IN SCOTLAND.

The authorities of the Northern Infirmary, Inverness, recently advertised for a charge night nurse, but they did not receive a single suitable application. In these circumstances the senior probationer is acting temporarily as charge night nurse. The staff usually consists of five charge nurses and seven probationers. There are sixty beds in the infirmary and forty in the fever wards.

### THE CRISIS AT CROYDON INFIRMARY.

A former nurse at Croydon Infirmary asks to be allowed to protest against the action of the Croydon Board of Guardians in trying to make such a rule as that referred to in our issue of October 20, to the effect that the certificates be signed by the medical superintendents instead of by the matron. She points out that without the matron's signature the certificate is of no value in the nursing world, and she inquires whether the Guardians realise the fact that if this rule is enforced, "they have been obtaining the services of the nurses under false pretences, as the understanding is that it is a training school with lectures given by the matron and medical superintendent." Our correspondent proceeds:—"By this rule the Guardians not only do a gross injustice to the present staff of probationers, but detract from a professional standpoint, from the value of the certificated nurses whom they have sent to all parts of the country. In the issue of our paper, The Hospital, dated October 20th, I read, 'The matron refused to sign three of the certificates because the probationers were not competent, but that the medical superintendent thought otherwise.' Surely the matron knows more of a nurse's character than the doctors, and in signing is responsible for such, not only in our nursing capacity, but as to our moral character, and if she considers any nurse likely to bring discredit on the school in which she was trained, I contend that she is justified in refusing to put her name to a certificate. I think that every nurse in the Croydon Infirmary, and many of those who have left, will be with me in sympathy for our matron, and against the Board of Guardians if they depose her from her position."

### NURSES' CLUB IN DUBLIN.

The inaugural meeting of the Nurses' Club in Dublin was held last week at the National Eye and Ear Infirmary, Molesworth Street. There was a large attendance, all the Dublin hospitals and nursing institutions being represented by their matrons. Miss Huxley, Lady Superintendent of Sir Patrick Dunn's Hospital, was unanimously elected President of the club; Miss Hampson, late Matron of the Rotunda, Hon. Secretary; and Miss Rae, Lady Superintendent of Cork Street Hospital, Hon. Treasurer. An executive and general committee was also formed. The objects of the club are to provide reading rooms and literature for nurses, to form classes and lectures on medical and scientific subjects, and where nurses may discuss any questions relating to their profession. The club rooms are situated in St. Stephen's Green. The project has been most warmly received by members of the profession.

### THE PAY OF IRISH NURSES.

Complaints are being made in the Dublin papers that nurses in Ireland are very inadequately paid; and we notice that, according to a private nurse in the capital, "the reason why trained nurses in Ireland receive such small salaries, in comparison with their sisters in England and Scotland, is that the public who employ them give such small fees." She maintains that nursing institutions do as well for their nurses as they possibly can under the circumstances. "They give as good food and accommodation as similar homes in England and Scotland, but are unable to give the same salaries, because, whereas two guineas a week are given in these countries, one guinea a week is considered adequate in Ireland." The difference is certainly very great, especially in the case of those Irish nurses who are as well trained and as competent as others. But it is not easy to suggest a remedy so long as the Irish public think a guinea sufficient. Moreover, in many instances, we fear that it is as much as they can afford to pay.

### NURSING AT NORVAL'S PONT.

A CORRESPONDENT, writing from Norval's Pont, says:—

"I can hardly believe it is the same place I came to a short time ago. Our enteric division developed to the extent of three or four marquees, and has dwindled again to one. Then we started a surgical marquee and a general medical division. We had more sisters up, and more still, in readiness to receive the commission which passed us by on its way to Pretoria. We have increased our staff of orderlies, having more than one apiece now: they seem to like waiting on us. I believe it exempts them from certain duties in the camp. What they all do is a profound mystery: we imagine that one peels the potatoes and another puts them in the pot, while a third holds the fork in readiness to see if they are cooked. Our mess is a very simple one—we do not go in for elaborate dishes. Common necessaries are not always to be had. We have had to go without butter or fresh milk for days, and eggs have been quite unobtainable except for the patients. However, we get on very well, and what we lack in luxuries we make up for in fun. Just now there is very little to do; we are striking marquees all round preparatory to taking over the Edinburgh Hospital. After living under canvas for over three months we do not altogether like the idea of iron buildings. It is nice to be able to draw back the sides and let the air play round your patients. In this climate in fair weather, I think it has been pretty well proved that patients under canvas do better than within four walls. Of course in driving rain patients' beds may have to be moved from the sides to the centre of the marquees to avoid the damp or occasional dripping, but my experience is that this can be done as a rule in time to prevent harm to anyone. As for dust, I do not find that it gets more into marquees than into buildings if proper precautions are taken."

### AN EXPEDITION TO BEGBIE'S WORKS.

A MEMBER of the Army Nursing Reserve Service, writing from No. 6 General Hospital, Johannesburg, says:—

"It may interest you to hear about an expedition one of the sisters and I have had with a friend to the scene of the explosion at Begbie's Works. The houses and buildings of one street are totally wrecked. The explosion occurred at the place where the Boers manufactured all their shells, and it is not known to this day whether the explosion was due to an accident or to wilful design. The men who were at work were so blown to pieces that no one knows how many lives were lost. Roofs have been torn off the houses, walls knocked down, iron girders and sheds are bent and crumpled up as if they were tinfoil. A great deal of the machinery still remains intact, though much is damaged, ruined, or useless. It must have been an awful explosion, and, judging by the different models of shells of all patterns, a great work must have been carried on for a long time. The ruins are quite one of the sights of this place. I have been hearing about the way our soldiers have to loot the farms. One regiment has just come back with quantities of cattle and poultry, also some horses and Cape carts. They are obliged to burn what they cannot bring away. It must be sad work doing this, and the men feel very sorry for the children, and leave a few mealies behind for them. Prisoners, too, are brought in every day. The "Princess Christian" train has just come in, and we were delighted to meet the sisters again, as we have not met since we left Wynberg. We had not much time to exchange experiences, but they told us they had been so fortunate as not to lose a single case on the train itself."

### DROGHEDA DISTRICT NURSING ASSOCIATION.

A VERY enthusiastic and representative meeting has been held in Drogheda under the chairmanship of the mayor, in support of the District Nursing Association. As to the work done in the past, it is sufficient to say that 8,880 visits were paid in the year by two nurses. This shows how thoroughly the nurses are appreciated, but it also proves the necessity of more nurses. One of the speakers observed that, "if the nurses left the town for a period of six months, everyone would understand what they are." But even if one fell ill for a short time, the people accustomed to her ministrations would suffer; and one woman ought not to have the enormous strain of between 4,000 and 5,000 visits imposed upon her. Therefore, we hope that the eloquent words uttered at the meeting will be followed by practical results of a satisfactory character. A concert is to be held on Monday week in the Whitworth Hall, Drogheda, in aid of the funds of the Association, and no doubt a substantial sum will be realised. But the best help that can be given is the regular subscription of persons of all classes.

### AN OIL STOVE FOR A DISTRICT NURSE.

IT cannot be expected that the ladies' committee of a district nursing association will never make mistakes, but the following case shows a deplorable lack of womanly consideration. We do not see why a committee of ladies should solemnly meet in order to discuss the burning question of whether the nurse is to eat her breakfast during the winter with an oil stove burning, or whether she is to have a fire. The question is one that might well be left to the nurse's discretion. In this instance, however, the question was not only discussed at length, but the committee decided that the district nurse, who is not only responsible for the poor in several villages, but is also required to act as midwife if any poor woman is in need of her services, ought to be satisfied on her return—perhaps after a walk of three miles in the rain or cold—to find an oil stove to welcome her, "because of the expense of coal!" This is pitiful economy with a vengeance, and we wish that the correspondent who sends us these facts had given us permission to name the committee which has manifested such a deplorable lack of good feeling. We are not surprised to learn that the sum of 12s. 6d. per week is all that the landlady with whom the nurse lodges receives for board and attendance, and that the daily bath is regarded as an unnecessary luxury.

### SUTHERLAND BENEFIT NURSING ASSOCIATION.

AT the half-yearly meeting of the Executive Committee of the Sutherland Benefit Nursing Association the Duchess of Sutherland presided. The first business was the consideration of the fifth annual report, which shows that 253 cases were attended by the Association's nurses during the year ending July 31st, 1900. There are now fourteen nurses at work, and the only discouraging paragraph in the report was the record of the death of Nurse Morrison from typhoid fever. The report having been read and adopted, the office-bearers were re-elected. The welcome announcement was then made that Mr. Carnegy, of Scibo, had given a subscription of £100, and Mrs. Carnegy £25. It was unanimously resolved to adopt a scheme, somewhat similar to that of the Presbyterian Church of Ireland for colporteurs, for providing pensions for nurses. All premiums are to be payable by the Association, and all policies are to be payable to it. A letter was read from the clerk to the Dornoch Parish Council, asking on what terms the Association could supply nursing for paupers. The Secretary and Superintending Nurse, Miss Stevenson, was instructed to issue a circular to Parish Councils, directing their attention to the scheme drawn up by the Association in 1896 for the provision of nursing for sick paupers.

### WATCHING THE C.I.V.'s AT ST. MARY'S HOSPITAL.

For the past week active preparations were carried on at St. Mary's Hospital for the return of the "C.I.V.'s." The temporary roof of the "Clarence Wing" was built upon, and accommodation for about twelve hundred people made. Notwithstanding this the secretary had to refuse hundreds of applications.

"The atmosphere inside the hospital itself," writes a nurse, "was a mixture between Christmas time and the relief of Mafeking. The children in the Crawshay Ward sang 'Rule Britannia' for days before, and made a noise quite beyond the ordinary work-a-day allowance, and even porters whistled the tune in the early morning as they carried along and emptied their coal-skips. The Bank Holiday element was at its very best in the casualty wards. Festivities commenced there on Friday night; the people just poured in, one woman very drunk; in time she got to the singing stage: she was accompanied by a man, who whistled in discords. The steward was early at work on Saturday morning superintending his porters as they carried out forms and chairs. When the official news came that the march past was put off until Monday it was a bit of a shock. There were the nurses on the very 'tiptoe of expectation'; but these little mental disturbances are only trifles after all, and no one was any the worse at the end of the day. At last the wished-for day came. The hospital looked very gay: flags at the top, flags from the windows, flags everywhere. All were in their places in good time, and all were prepared to rejoice. As for the students, when the procession came in view they shouted, and shouted, and shouted until the last bit of khaki passed out of sight. The C.I.V.'s seemed to thoroughly appreciate their reception: some smiled and waved back, while others did not seem strong enough for enthusiasm."

### THE NURSES ON THE "AURANIA."

There were two nurses only on board the *Aurania* with the C.I.Vs. One was Sister Pope, who was in the hospital at Kimberley during the siege; and the other, Sister Pugh, who was on the staff of the Welsh Hospital at Pretoria.

### NEW NURSING HOME AT HANLEY.

A nursing home, a memorial of the Queen's Diamond Jubilee, has just been opened at Hanley. The house selected by a sub-committee of the Hanley Nursing Society for the purpose has been very much altered and improved since it was purchased. The accommodation provided is excellent, and owing to various gifts, including one of £200 by Mrs. Mason, it is entered upon absolutely free of debt. The need of the institution is apparent from the fact that 500 cases are attended to by the nurses annually, and between 5,000 and 6,000 visits made. Hitherto there have been only two nurses, but the opening of the home will, it is hoped, be followed by an addition to the staff.

### "GOOD STRONG GIRLS" AS NURSES.

In most respects the annual report of the Cumberland Nursing Association affords matter for congratulation. It has but been in existence three years, and there are now 25 nurses working in connection with it. But of these only six are fully-trained nurses, the others being village nurses trained hitherto for six months only. The term has just been increased to nine months, and in future all village nurses will be trained at the Plaistow Training Home, which is affiliated to Queen Victoria's Jubilee Institute. In one district two Queen's nurses are employed, and the committee are doing their best to make the advantages of having a Queen's nurse in others. Lady Lonsdale said at the meeting of the Association that the committee "found rather a difficulty in getting Cumberland lasses to come forward for training as nurses," and she appealed to ladies "to try and find good strong girls for the purpose." The "good strong girls" may do as a makeshift, but the aim of the Association should be to obtain sufficient funds for the employment of an adequate fully-trained staff.

### NURSING AT ABERYSTWITH.

The first nurse of the Aberystwith and District Nursing Association began work on October 21st, 1899, and the second on January 23rd, 1900. We agree with Mr. H. C. Fryer, who presided at the annual meeting of the Association, that it was a scandal that something had not been done in that direction before in such an important and flourishing town as Aberystwith. But better late than never. In commenting upon the report, which showed that between October 19th last year and September 30th, 1900, upwards of 4,000 visits have been paid, Prebendary Williams said that "before the Society was formed persons went to certain of the sick poor with delicacies and money every week and left, perhaps, a more deserving case next door out in the cold. This was now happily done away with, and all sick and needy were readily attended to." No better testimony to the value of the services rendered by the nurses could be afforded. During the present year Miss Frank, the inspector of nursing from the Queen's Jubilee Institute, visited the town, and her report upon the work was very satisfactory. We observe, with pleasure, that there is a strong working committee, on which representatives of all religious bodies are represented.

### A YEAR'S EXPERIENCE AT SOMERCOTES.

It is just a year since the formation of a Trained Nurse Fund was first mooted at Somercotes, and a nurse has been in readiness for some months. The movement had to encounter many discouragements and difficulties at the outset, and the necessary money to justify a start was very hard to obtain. But at the first annual meeting the other day Mrs. Hicking, the secretary, had a satisfactory report to make. The nurse since her arrival has attended 43 cases and paid 1,223 visits. As she has not yet had experience of the more trying winter months, it is evident that there will be plenty for her to do. Happily, the excellence of her work has created an impression, and Mr. James Oakes, who had already contributed £10, has promised a similar sum for two years more, while others who were doubtful at first now freely admit that Somercotes has gained an institution it would not like to part with.

### SHORT ITEMS.

On Sunday the annual Harvest Thanksgiving Service was held at St. Mary's hospital. The service was conducted by the chaplain, assisted by the Rev. W. Whalley (former chaplain of the Hospital), who preached a suitable sermon. The choir was composed solely of nurses and students. The decorations were very artistic. The home sister (Miss Maddison) and the night sister (Miss Whitson), assisted by the nurses, spared neither time nor labour to make the little chapel look beautiful.—At the fifth annual meeting of the Alyth Nursing Association, presided over by Sir James Ramsay, it transpired that in place of the deficit shown last year there is a balance to the good for the last twelve months of £81 13s. 3d. This is an exceedingly satisfactory change, as one of the speakers said, and indicates high appreciation of the nurse.—A concert and entertainment at Redditch the other day on behalf of the Redditch and District Nursing Association realised the respectable total of £33 3s., which has been handed over to the treasurer.

### Lectures on Medicine to Nurses.

By H. A. LATIMER, M.D. (Dunelm), M.R.C.S. Eng., L.S.A.Lond.; Consulting Surgeon, Swansea Hospital; Past President of the South Wales and Monmouthshire Branch of Brit. Med. Assoc.; Past President of the Swansea Medical Society; Hon. Life Member of the St. John Ambulance Association, &c.

### THE FACE AS AN EVIDENCE OF DISEASE.

IN the matter of the face as an indication of disease you will notice, first of all, that the colour varies very much with the supply of bright-coloured arterial blood to its skin, or with congestion of dark-coloured venous blood which occurs when a retarded return of the same to the heart takes place, as the result of any impediment to its passage through the lungs and through the heart. When arterial blood is present in a part in a larger quantity than is proper, it is customary to call the resulting congestion an *active* one; when venous blood is similarly present in excess, we speak of there being a condition of *passive* congestion of a part.

We may take as an example of active congestion, as exhibited in the face, the florid complexion of a stout plethoric person; this generally occurs in stout men who appear to be in robust health. In the old bleeding-days such a person would have been regarded as suffering from a liability to inflammatory attacks of internal organs, or to being in danger of an apoplectic attack, and he would have been bled from time to time to ward off these possible events taking place. Our ancestors lived in a chronic dread of what might happen to them, and took great thought for the medical to-morrow; consequently they were continually dosing themselves with medicines—a habit which, alas! their descendants have not by any means departed from, if we may judge from the wealth acquired by quack pill and medicine vendors—being bled, and having setons and "issues" made in their bodies. It was customary for people to present themselves to their medical attendants in the spring months so that they might be bled; those who did so imagined that they had a plethora of rich fluid within them which it was necessary to reduce if they were to maintain their health. After the bleeding had taken place I suppose they left the doctor's surgery feeling, with the poet, that "their former richness was but plethoric ill." But as it is not with the ruddy-faced person that you will, as a rule, be concerned, I will proceed to speak of those variations of complexion indicating ill-health with which you will be brought into contact. Foremost of these invalids, as being perhaps the most frequently met with in practice, are those who are suffering from jaundice. Here not only is the skin generally tinged with a yellow hue, but the whites of the eyes are similarly stained in a very characteristic manner. Indeed, the staining of these usually white tissues often precedes that of the skin, and is even a marked feature of the jaundiced state when the skin, from its more abundant supply of blood, hardly shows any discoloration. I need scarcely tell you that generally this staining of the body, in jaundice, is due to the circulation within the blood of the colouring matter of bile, which then escapes from that fluid during its transit through the body and gives its peculiar tint to the parts in which it lies. I say "generally" when giving this explanation of the matter, for the jaundiced hue is not always due to bile coloration, there being some cases when it would appear that the yellowness of the skin and eyes is due to their being stained by colouring matter which has escaped from the red-blood cells which have been destroyed by some one or more diseases which have this effect upon them. Such points, however, are matters of the deeper science of pathology with which I need not trouble you. It will be of more interest if I point out that the shades of yellowness you will meet with differ in different persons according to the pallor, redness, or lividity of their complexions. In dark-complexioned people, for instance—such as brunettes—their jaundiced faces take quite an olive-coloured tint; in pale-faced folk the skin and eyes have the hue of the lemon imparted to them; and where there is a blueness of lividity due to impediments in the circulation of the blood, or to the circulation within the blood of dark-coloured, vitiated bile, the consequent tint may be quite of a dark-green or blackish variety. Such details as these may interest you. If you are observant of things in your daily work, and have noticed such varieties of jaundiced faces as I have been speaking of, you will be glad to find that reasons are forthcoming for the facts I have placed before you.

Now let us turn to conditions of the face which are the opposite of those I have just been discussing. Then I was drawing your attention to excesses of colour: now I call you to observe cases where there is a deprivation of the same. The disease termed anæmia affords us a ready example of this state of pallor; for when this is extreme the face, the lips, the gums, the tongue, and the inside surfaces of the eyelids are pallid to a marked extent. There are varieties of this disorder which we call anæmia, due to more or less grave disturbing causes, and I presume that I shall be enlarging on these topics when I arrive at their consideration at an appropriate time, in a later part of these lectures, when diseases of the blood will be under review. Suffice it to say now that you will constantly meet with cases of simple anæmia among your girl friends, in young adult life, for there is a peculiar liability to this disease in members of your sex who are at the period of adolescence. The cause of the absence of colour I may, however, allude to now. It arises from a diminution in the number or in the colour of the red-blood cells in the fluid coursing through the minute capillaries of the skin and mucous membranes, as elsewhere. The skin and mucous membranes are open to observation, so you can very fairly gauge the amount of deterioration of the blood which has been going on in the anæmic person; of course, you will understand that the same deficiency of red-blood cells exists everywhere in the blood, and that the deeper-seated tissues are as pale as are those on the surface. Inasmuch as the red cells of the blood are the carriers of oxygen all over the body, this absence of them causes a serious condition of impaired vitality to obtain; for if the processes of oxidisation do not go on within us we cannot maintain our heat, and effete and used-up materials there cannot be changed in such a way that they can be removed from the system; so anæmia, from any cause, calls for speedy remedying if health is to be maintained.

Another condition in which extreme pallor often results is that of disease of the kidneys, accompanied by the appearance of albumen in the urine, and by dropsy. Here there ensues a peculiar white face with swellings of the looser textures on it: such textures are most marked at the eyelids, and it is there that the dropsy from which the patient is suffering especially reveals itself. A face of this sort once seen impresses itself upon your memory, and remains there as a mental picture for all time. I saw such a one a few days ago—a pasty, white, puffy face, with bulging, translucent bags underneath the eyes—and immediately my mind recalled other such cases I had seen. The mention of this puffy face reminds me that it is not only in the matter of coloration there that disease shows itself, but that fulness or emaciation prevail in this part as in others. Dropsy, of kidney origin, generally first shows itself in puffiness of the eyelids; for there the tissue is of an especially loose texture to allow of free action of the eye-ball in its socket, and so water readily collects in its meshes and distends them.

(*To be continued.*)

### Nurses and Orderlies in South Africa.

By a Correspondent.

### A CHAT WITH TWO OF THE SISTERS.

Nursing Sisters from South Africa are such rapid birds of passage that it is difficult to secure them for a few minutes' talk; however, late one evening last week I was able to catch two who had arrived in the *Dunera* hospital ship.

### The Temporary Hospital.

"What happens," I asked, "to a transport when it is turned into a temporary hospital ship?" "The lower decks are removed," they replied, "and the space is fitted up as wards. The *Dunera* has five wards, four being as it were on one floor and one below. The wards are separated by iron doors, so that they may easily be shut off in case the vessel should threaten to sink, or ship too much water; they lie the length of the ship, with a passage between, so that the hospital takes up the entire ship's bottom."

"And what are the beds like?"

"In the two acute wards—the acute medical and acute surgical, as you may say—there are swinging cots, with room to pass between; but on the other side, where the convalescents are, the bunks are one over the other as in a cabin. This would not do for serious cases, but it is quite suitable for the men who are strong enough to be on deck all day. There are two decks: one for the men and the other for the officers and sisters. We brought no passengers."

### The Nursing Sisters.

"How many sisters were on board?" I asked.

"Sister Hoadley of the A.N.S., and Sisters Ferguson, Gibbs, Hobbs, Snell, Wilson, and Wood, of the Army Nursing Reserve. There was a sister for each ward; Sister Ferguson had the surgical ward and the operating theatre."

"Were there any serious operations?"

"One man had his leg amputated; we thought it would be necessary to remove another man's hand, but it healed up in a wonderful way during the voyage. We had no deaths, but one of our patients died just before we left Durban."

"Did the men benefit much by the voyage?"

"Yes, remarkably so; the sea air and rest did them a great deal of good."

"How many men were in the surgical ward?"

"The ward had forty-two beds, and four of these were empty. There were one or two medical cases also in the same ward. Some of the papers, by the way, have understated the total number we brought home; a good many disembarked at Plymouth, while we came on to Southampton, and this probably accounts for it. The total would be about 300."

### Details of the Nursing.

"At what hour did the sisters go on duty?"

"We went down to the wards at 7.30, and saw to the men's breakfast, and to any bad cases, and made any beds that were necessary. Then we left the men to their meal; our own breakfast was at nine, after which the morning's duties really began. We did dressings, took temperatures, and gave morning medicines, &c. In the surgical ward there was a good deal of work, and after the doctor's visit, Sister Ferguson was kept pretty busy until midday, after which there was less to do. We lunched at one, and were free for the afternoon unless any special case required us. At six we gave evening medicines and took temperatures, and we were supposed to dress for dinner just before seven—of course there was no compulsion about this."

"After dinner you were free?"

"Yes, except for the acute cases, which required dressings twice a day; these were attended to from eight to nine."

"Did you give massage?"

"Yes, a little."

"Does the sister superintend the giving out of linen, etc.?"

"No; she asks for sheets when they have to be changed. That matter is in the charge of the ward master, and he and the orderlies see to the food also."

"You were not present at the men's meals?"

"We were not; and sometimes we were puzzled at sudden changes or relapses in the state of our patients, which could only be traceable to the habit of sharing food, or bribing orderlies to give that which is unsuitable. A great deal of mischief is done in this way, and both doctors and nurses are frequently at a disadvantage through not knowing what a patient has eaten on the sly."

"Was the ship's food good?"

"Yes, excellent; the ship's people are of course accustomed to catering for large numbers, and they provided very well. There was a great deal given also, so that the men were well nourished."

### A Vexed Question.

"Do you think the men would rather be nursed by sisters, or by orderlies?"

"When they are very ill, they beg you not to let the orderlies touch them; they are not gentle in lifting, and they do not know how to make a sick man's bed. But when they are strong enough to swear at an orderly, they prefer him!'

"Have you had any experience of the St. John's Ambulance men?"

"Yes," said one of the sisters; "and personally I prefer them. At Estcourt I always asked for them for my ward."

"Because of their training?"

"Because they are better educated, and more refined than the average orderly."

### Under Canvas.

I enquired whether either of the sisters had been through a siege?

"We arrived in South Africa," they replied, "on the day that the siege of Ladysmith was raised; we were stationed at Estcourt, with No. 7 General Hospital."

"That is, the field hospital?"

"Not the field hospital proper; the sisters are not allowed there; it is on the fighting line, and the men are brought to it straight off the field. Their wounds are attended to by the doctors or R.A.M.C. men, and they are kept perhaps for a couple of days, after which they are sent to the General Hospital, and thence to the Base Hospital, where they stay until they are sufficiently recovered to be brought home."

"Had you plenty of food for the patients at No.7?"

"We were never cut off from supplies, though the line was occasionally tampered with, and sometimes there was delay, as the medical stores had to give place to the transports. We were on army rations, but when possible we bought food from the blacks, for the sake of variety, and when rations were low. It is such a treat to come back to English food! The beef, especially, was very tough in Natal."

"Trek ox?" I suggested.

"Yes. But our greatest want was fresh milk for the men. When they are very bad with fever they sometimes cannot bear tinned milk, even the kind called "Ideal," which is unsweetened. Occasionally we were able to buy fowls and eggs from the blacks, but they were very dear. One great advantage was that we were close to a river, and this, in a country full of insect pests, is an enormous boon."

### Royal British Nurses' Association.

THE LATE SIR HENRY ACLAND.

THE quarterly meeting of the General Council of the British Nurses' Association was held on Friday at the rooms of the Medical Society. Sir Charles Gage Brown was in the chair, and in his opening remarks he regretted the absence of the President, H.R.H. Princess Christian, and of the Hon. Treasurer, Mr. Langton; the latter being at Harrogate, attending an important meeting in the interests of the Association.

After the reading and confirmation of the minutes of the last meeting, the reports of the Treasurer and of the Executive Committee were read and adopted. The total receipts for general purposes since April were £348 11s. 1d., while the total expenditure was £415 17s. 10d. The bank balance was £45 5s. 9d., and the liabilities £125. The expenses were chiefly for the printers' bills, and it was explained that the autumn quarter was always unfruitful in subscriptions, and that the expenditure was usually at this time of year in excess of the income.

The Medical Hon. Secretary, who in the Hon. Treasurer's absence moved the adoption of both reports, said the members would be glad to learn that upwards of £80, besides gifts in kind, had been received since April for the Nurses' Settlement Fund, and there was every reason to believe that the scheme was being warmly taken up. The Executive Committee's report stated that during the last three months 93 nurses had been registered, 94 new members had joined the association, 15 members had withdrawn, and five had died. The death of Sir Henry Acland was a great loss to the association. It had been arranged that the usual conversazione should take place in December, and reference was made to the formation of various county centres of the association. Mr. Farden thought that there was great cause for congratulation, and that the steady wholesome increase in membership showed that the association was extending its influence; he referred more especially to the formation of the branch in South Australia. Speaking of the death of Sir Henry Acland, he said that Sir Henry had from the first taken a warm interest in the association. He had felt that nurses ought to have some public recognition, and he did what he could to assist the Association, which always had his warmest sympathy. A cause for congratulation was the removal of anxiety as to the health of one of the Vice-Presidents, Her Majesty the Empress Frederick of Germany; for many days her life had been considered to be in danger, but this anxiety seemed at any rate for the time to have passed away. Mr. Farden also alluded to the Auxiliary Nurses' Society—formed to materially assist members whose age prevented their joining the Chartered Nurses' Society, and which had met with marked success. After a great deal of work and trouble, it was hoped now to begin the work of this Auxiliary Society in December, and arrangements had been made for the office to be in the same building as that of the parent society. A lady had already been selected to fill the post of secretary; she had been a trained nurse, and there was every reason to believe that the work would prosper in her hands.

Dr. Bezley Thorne, speaking on the report, reminded the Council that five years ago, when the annual meeting was held at Oxford, Sir Henry Acland took a prominent part in the proceedings. It would, he said, be impossible to describe his solicitude in making the meeting a success; the interest he took and the knowledge of detail which he showed were astonishing. Personally, he should always feel grateful for Sir Henry's interest and sympathy with the objects of the Association, of which at that time he (Dr. Bezly Thorne) was secretary.

Mrs. Dacre Craven said that Sir Henry Acland had taken the deepest interest in the higher education of nurses long before this Association was started. He looked forward to the day when nurses would take their places as professional women, as their brothers might as medical men; and she believed that an association like this would do more than anything else to bring about this result.

On the motion of Miss Thorold, seconded by Mrs. Dacre Craven, it was agreed that the appreciation of the meeting should be conveyed to Lady Acland.

After a brief discussion on the proposed bye-laws for the Australian branch, the proceedings were brought to a close.

### Enlargement of Haggerston and Hoxton Nursing Home.

THE new rooms at the Haggerston and Hoxton District Nursing Association's Home were opened on Thursday last week by Lady Florence Cecil. The proceedings began with prayers offered by the Rev. A. Tanner, Rural Dean of Shoreditch, after which the chairman, Dr. Hewitt Oliver, gave a brief outline of the history of the Association since its opening, under Miss Wells, eleven years ago. Beginning with three ladies, two of whom were nurses, and the third the tenant of the house, the institution had grown to its present proportions; and the accommodation was now sufficient for the matron and fifteen district nurses to live under one roof. There was still a debt of from £300 to £400 on the enlargements. It is hoped that the Bazaar to be held at Shoreditch Town Hall next May will help to pay this off. During 1899, 36,000 visits to 1,500 patients were paid, at an average cost which, he thought, was lower than other institutions had found possible to make it, i.e., 5½d. per visit. Both the chairman and subsequent speakers alluded to the harmony between the Association and the local medical men; there had been no friction during these eleven years, a fact which spoke volumes, and proved, first, the necessity of the institution in the district, and secondly, the thoroughness, discretion and efficiency of the nurses.

Lady Florence Cecil, Vice-President, said: "I have much pleasure in declaring the addition to this Nursing Home open, and I hope that the comfort of the nurses will be materially increased by the enlargement, and that consequently they will be able to carry on their work with even greater success than hitherto."

A vote of thanks to Lady Florence Cecil was moved by the Rev. A. Peile, Master of St. Katherine's, seconded by Sir William Broadbent; both paid high testimony to the work of the Association. Sir William alluded to the direct benefit, not only to the poor, but to those of limited means; and spoke of the good effects in the homes visited by trained nurses.

The Rev. Dacre Craven having also spoken, tea was handed round by the nurses, and visitors were invited to inspect the additions. These consist of the room in which the meeting was held, four bedrooms, two bath rooms, and half the nurses' sitting-room, which has been enlarged to double its former size; it now makes a comfortable room in which to sit when the day's work is done. A friend has given a piano. The former box-room downstairs has beent urned into a convenient "District Room." Among the guests were Miss Peter (Q.V.J.I.), Miss Matthew (Head of District Nursing Home, Camberwell), and others, with many of the local clergy.

### The Nurse in Hot Climates.

By EDWARD HENDERSON, M.D., F.R.C.S. Edin., late Surgeon, General Hospital, Shanghai.

(*Continued from page* 55.)

*Mosquito Nets and Mosquito Houses.*—Mosquito nets, which have usually a border of thin cotton cloth, are either secured by being tucked in under the mattress, or they surround the bed the lower border resting on the floor; in the last case the border is weighted, usually with small shot, so that the position of the net remains fixed. During the day the net is raised, and the bulk gathered up conveniently on one side or on the top of the bed. Before the net is lowered and secured for the night the attendant should make sure that no mosquitoes are concealed in its folds or in the space which it encloses, including the space under the bed when a weighted net resting on the floor is used. The material of which mosquito nets are made is, of course, very easily torn, and it is astonishing through what a small hole a mosquito determined to effect an entrance will find its way. It is the nurse's duty to see that the curtain which protects her patient is not in any way damaged. The mosquito house is simply a framework of wood over which mosquito netting is tightly stretched; it is entered by a door at the side, and is usually provided with a punkah, which hangs from one of the cross bars in the roof and is pulled through a hole in the framework. Other modifications—such as that in which a wooden frame, with uprights only at the corners, supports a net weighted along its lower border—need no special description; details may vary a little but the principle is the same in all. As time goes on it seems not unlikely that wire gauze windows and doors will largely take the place of mosquito nets.

Enough has been said regarding the general principles which govern nursing in hot climates, and sufficient details have been given regarding their application by the nurse. Attention must now be directed to a few of the principal diseases for which the nurse's services will be required, and to any duties connected with these which seem at all unusual or peculiar.

*Bowel Disorders.*—One of the first things likely to strike the nurse who is new to the East is the large proportion which bowel disorders bear to other medical cases. That heat is an important factor in the production of these cannot be doubted, though it might be sometimes difficult to say in what way exactly it had acted in individual cases. The mucous membrane lining the alimentary canal may suffer in hot climates in sympathy with the skin with which it is continuous; the general enfeeblement which tropical heat produces in Europeans is by no means confined to the muscular and nervous systems, but through these seriously affects the functional activity of the digestive organs; heat causes rapid decomposition, making ordinary food unwholesome; heat favours the rapid growth of specific germs such as those on which cholera and the epidemic forms of diarrhœa and dysentery depend. Explain the matter how we may, the fact remains, and is abundantly proved by the records of our own registrars in England, as well as by the medical history of communities in the sub-tropics, where the diseases which accompany periodic and decided variations in temperature can perhaps best be studied. In all cases where the bowel secretion is affected, special attention must be given to the motions, their frequency, character, and the immediate effect they may have on the patient. As the nurse is more or less constantly in the ward or sick-room much of this duty will rest on her, and in the case of female patients she will be chiefly responsible. In recording the number of the motions distinction should be made between those passed during the day and those passed during the night. A patient will go to stool during the day as the result of slight disturbance which would have little effect at night, and on this account motions passed during the hours usually devoted to sleep may be taken to indicate a more serious form of the disease under treatment. Should the nurse be asked to describe the motions she should do so systematically, having regard to odour, quantity, consistence, colour, and especially to the presence of any extraneous matters as blood mucus, pus, or worms. The colourless rice-water stool of cholera, and the scanty motion containing blood and mucus seen in dysentery, are easily recognised; the great exhaustion ending in collapse which follows the first, and the pain and straining which usually accompany the second, further distinguish the cases. During the first few hours, occasionally during the first few days, there may be nothing to distinguish a case of cholera or dysentery from one of simple diarrhœa; the nurse should bear this in mind, although her services will seldom be required before a definite opinion as to the exact nature of the illness has been expressed by the physician in attendance. In cases of bowel disorder of any severity the use of a bed-pan is necessary; in most cases of dysentery it should be insisted on by the nurse. If the bed-pan is refused by the patient, as it often is in the beginning of an illness, a suitable receptacle must be placed at the bedside. Nothing can be worse in cases of diarrhœa or dysentery than visits paid to a cold or exposed lavatory during the night. Bowel washing is often ordered, either as a preliminary step to the administration of an opiate or nitrate of silver enema, or simply as a routine treatment when it is thought necessary to remove irritating secretions from the lower bowel. Plain water at a temperature of 100° Fahr., or better still, water which has had its specific gravity raised by the addition of 0·6 per cent. of common salt, roughly speaking, a teaspoonful to the pint, should generally be used for these washings; but occasionally boracic acid or some other suitable antiseptic, an astringent, or bicarbonate of soda, is ordered to be dissolved in the water. The giving of these enemata is an operation frequently entrusted to the nurse. An irrigator is the proper instrument to use in washing the bowel, the object being the production of a gentle, continuous and easily regulated flow, for which the enema syringes in common use do not sufficiently provide. The irrigator should be fitted with about five feet of indiarubber tubing ending in a nozzle suited to the case under treatment; the common bone nozzle, two inches long, will be sufficient in ordinary cases, but in some gum elastic is a better material to use than bone, and the length of the mount may be increased with advantage. The introduction, or at least the control, of the nozzle can often be left to the patient, while the nurse stops the escape of the water by using the stopcock, or simply by pinching the indiarubber tube between her fingers; if this is done after a little water has first been allowed to escape, both nozzle and tube remain full, and the trifling inconvenience of introducing air into the bowel is avoided. As the force employed depends on the height of the reservoir, care must be taken in raising this to avoid undue or sudden increase of pressure; two or three feet above the bed will be found a sufficient elevation in most cases, and the lowest level which allows of a slow continuous flow is the one generally to be preferred.

(*To be continued.*)

### Echoes from the Outside World.

AN OPEN LETTER TO A HOSPITAL NURSE.

OWING to the untoward fact of the *Aurania* being detained by bad weather, the arrival of the C.I.V. seems to have been divided into two days. I suppose that people who were actually in London may have heard the report of the non-arrival of the vessel on Saturday in time to prevent them starting out, but those in the suburbs and the country had, of course, nothing to guide them, for even telephone and telegraph offices were besieged in vain. So the streets of London were filled with sightseers who had been disappointed of their promised show and had turned into the thoroughfares to get what satisfaction they could from the decorations. In most places these were exceedingly good, although there was naturally a sameness in the styles. This, however, was as it should be, for if every house had tried a scheme of its own, the effect would have been far from delightful. Still, I felt grateful to the proprietors of the "Daily Graphic,' who had made a charming arrangement of white with waving pampas grass on both sides, relieved with pink silk and gleams of silver. But I was quite sorry for the Griffin at Temple Bar: he seemed to feel his position so acutely. Around the base of the stand were evergreens and growing plants which were nice enough, but above from the corners to the lamp posts were ropes of paper flowers. I conclude that the notion had been to use red, white, and blue, but either the wreather was colour blind, the supply of the right shade of colour had given out too soon and others had been substituted, or the rain had made the colours run. Anyhow, the result was here a red rose, there a pink one, now a blue flower, next a purple, next a heliotrope, with white, cream, and yellow plentifully intermixed. As I heard a man near me remark: "I 'spect the City thought they'd lay the colours on strong to make up for the khaki and the veldt." Perhaps so.

AFTER the show on Monday the feeling which was uppermost in the majority of minds must have been wonderment as to what London would do when "Bobs" came home, since the arrival of the C.I.V.'s had caused such a monstrous demonstration, and had brought together such an unruly crowd. That London officials can organise a large crowd, and avoid flagrant disorder and serious loss of life, was shown at the time of the Jubilee, but then the war-fever was not upon us. When the men of the *Powerful* came to the Metropolis the excuse was made that the authorities never dreamt of so large a crowd. The same plea could not hold good on Monday. Personally I had no inconvenience; our seats in the Strand were close to Wellington Street, and we were in our places early; but the mob below, consisting largely of the roughest element south of the Thames, made one almost sick to watch, especially when here and there gleamed the white face of a frightened baby or a fainting woman. Had it not been for the ambulance men and the Red Cross nurses, aided by Volunteers, the loss of life would have been far greater. They worked splendidly, and it was grand to see an inspector of police rescue a woman once. She was down in the crowd; the people around could not clear enough space to get her on her feet again. The man, seeing the situation, charged in with the horse to the spot, and, amidst cheers, brought the poor thing out with him. As to the procession, the first part was distinctly inspiriting, but then the men came in twos and threes, as the surging crowd let them through, many of the C.I.V.'s looking tired with the struggle. The enthusiasm was marvellous. I reserved my loudest cheers, first for the captured Transvaal flag, and then for the nurse who sat on the box of one of the invalids' carriages between two soldiers. I was so glad she was there to represent her sisters.

AMONGST the speakers at the National Union of Women Workers, Mrs. Creighton was one of the most interesting. She chose as her subject the need of a purpose in life, and referred to the time when the one aim, not only of every girl, but of all her sisters and her cousins and her aunts had been to get married. Now matchmaking was considered vulgar, and the question of marriage was often totally ignored in considering a girl's life; and what had taken its place? The most dignified name she could give to this new object in a girl's life was the desire to "have a good time," and she wondered how much this was an improvement on the old object. The Bishop of London's wife went on to pay her husband the compliment of saying that "personally she was very much in favour of matrimony," because she believed "the single life to be the more difficult, and rare gifts of unselfishness were needed to prevent the character suffering from it." But she also dwelt on the work to be done for the service of mankind, which only the single man or woman could do, and my thoughts flew involuntarily to the nursing profession. She addressed very nice words to those who were conscious of a thread of purpose running through their lives, and put in a plea for some who took life so seriously that other young people called them prigs. She admitted she was inclined to believe that all people who were worth anything had to go through a period of priggishness, and they must take it as they would the measles, and get it over quickly. Mrs. Creighton's concluding remarks to all were a little sermon in themselves. She alluded to the heavy responsibility which rested on the young. "If they would use their liberty only to amuse themselves, to have a good time; if they neglected to hear the call to service, and fit themselves to bear their share of the world's burden, then women would miss the great opportunity which was now theirs of becoming fellow-workers, fellow-citizens, with men, and of learning how to do that work which, in the great economy of the world, could only be done by them."

THE death of Professor Max Müller was not unexpected by his friends, but the knowledge that no more will ever be written by him must bring a feeling of sadness to many who never knew the great man save in the spirit. It is as the author of "The Sacred Books of the East" that Max Müller is perhaps best known, but no writer of his time has been more prolific. Books, pamphlets, magazine articles, letters, and lectures flowed from his brilliant pen in rapid succession, whilst he translated, edited, and lectured, either in English or German, with consummate ability. As an expositor of comparative philology and mythology he was said to be unmatched.

TO most of this generation Sims Reeves is merely a name, because, notwithstanding that even at the time of his death engagements were being booked for him, he had seldom appeared in public of late. But this is hardly to be wondered at considering that he celebrated his 82nd birthday last September. I remember going to a concert at which Mr. Reeves sang about twelve years ago, when, of course, he was 70 years of age, and though there was no real voice left, I could not help being marvellously impressed with the beauty of the phrasing and the enunciation. The song he was rendering was an old favourite which I must have heard dozens of times before, but I came away feeling that whereas before I had only listened with my ear, this time I had listened with my soul. Mr. Reeves was a strong advocate of the necessity of singers being "careful livers," and he made a rule of only taking two meals a day, breakfast at eleven, dinner at four. After the performance was over, before retiring to rest, he would indulge in a couple of lightly-boiled eggs. Mr. Sims Reeves married for the second time five years ago, and leaves a widow and infant child.

### Examination Questions for Nurses.

### INGUINAL OR FEMORAL HERNIA.

### Result of October Competition.

The question was as follows:—"In a case of Inguinal or Femoral Hernia (in private work) what steps should you take to alleviate suffering pending the doctor's arrival?"

### No Prizes Awarded this Month.

It is regrettable that the answers sent in to this simple but important question should not reach a sufficiently high standard to merit a prize in any instance. It is a serious thought, and should give food for reflection, that so many inefficient nurses (judging from their papers) are at work in the world. One can only hope that carelessness in reading the question and slovenliness in expressing themselves counts for something, and that they are really better nurses than one could gather from their papers.

### The Two Best Answers.

Nurse Edith and Nurse Nora's papers are by far the best, though each one fails on some important point. Nurse Edith does not specify any particular method of applying ice, and Nurse Nora speaks of the application of cold in a way that would soon cause it to become a species of poultice.

### Principal Faults in the Papers.

It is astounding to read of the brisk treatment advocated by some nurses. The question does not mention that in many cases, in most indeed, where a nurse's aid is called in the hernia is strangulated. The wording was expressly chosen to draw out the nurse's knowledge of the symptoms present in the irreducible but not necessarily strangulated and the strangulated, and therefore irreducible. Reducible herniæ do not, as a rule, give much pain. These nice distinctions are quite overlooked in almost all the answers. Several writers advocate a hot bath, and one boldly recommends trying taxis preceded by "an aperient"! This candidate has indeed much to learn, for she mentions this treatment as to be tried if "a delay of a few days" may occur before the arrival of a doctor! In such circumstances a patient with a strangulated hernia would probably be dead without any assistance from the efforts of his over-zealous attendant.

Taxis requires the most perfect knowledge and the lightest hand. Where many experienced surgeons hesitate to attempt it nurses would do well to imitate their discretion.

Some papers, again, recommend an enema—in cases of strangulation most dangerous, and in *no* case to be employed without direct orders.

Nurse Foster sends in a fairly good answer (she has formerly been a prize-winner), but is dangerously incorrect in saying that a poultice might be applied. It would not hurt in an ordinary irreducible hernia, though of doubtful benefit; but with one that is strangulated it would have most serious, and perhaps fatal, effects. She has some doubts herself as to the wisdom of the step without a doctor's orders.

This question will be repeated later in the season. In the meantime I hope that nurses will thoughtfully consider the matter. I will say nothing more now, because it is desirable that study and experience should dictate answers, and not that they should be made too easy by copious instructions from the examiners.

### Question for November.

State what you know of the best means of nursing plague, what steps you would take to avoid the spread of infection, and what measures you would adopt to insure personal immunity whilst ministering among the sick.

[It has been thought by the management that it might be of general interest and advantage for nurses to study the plague question. Kindly note what I am about to say. It has always been a cause for grave displeasure when competitors in these examinations have drawn their answers even partially from text-books, the object being to strengthen a nurse's powers of observation and to draw upon her reserve of practical effort and retentive memory. In the present question, however, with regard to plague personal observation and knowledge are in most cases absent; not more than one nurse in 3,000, perhaps even fewer than that, has had any real acquaintance with the dread disease; therefore it is permissible for this one occasion only to study papers and handbooks. I shall be obliged to any nurse who has worked amongst the plague-stricken, and who sends in a paper if she will mention the fact in her answer. The Examiner.]

### Rules.

The competition is open to all. Answers must not exceed 500 words, and be written on one side of the paper only. The pseudonym, as well as the proper name and address, must be written on the *same* paper, and *not on a separate sheet*. Papers may be sent in for fifteen days only from the day of the publication of the question. All illustrations strictly prohibited. Failure to comply with these rules will disqualify the candidate for competition. Prizes will be awarded for the two best answers. Papers to be sent to "The Editor," with "Examination" written on the left-hand corner of the envelope.

N.B.—The decision of the examiners is final, and no correspondence on the subject can be entertained.

In addition to two prizes honourable mention cards will be awarded to those who have sent in exceptionally good papers.

### Resignation.

The Matronship of Marland Hospital, Rochdale.—We understand that Miss Bellamy has resigned the matronship of the Marland Hospital, Rochdale, in consequence of her approaching marriage with Mr. S. Turner, of the well-known firm of Turner Brothers, Rochdale. Miss Bellamy has received many tokens of the regard and esteem in which she is held by the nursing staff and servants of the Marland Hospital.

### Appointments.

Fulham Infirmary.—Miss Mary Maggie Beales has been appointed superintendent of night nurses. She was trained in the same institution.

Lochart Hospital, Lanark.—Miss Mary Macdonald has been appointed matron. She was trained in the Sunderland Infirmary and has since been staff nurse for two years in a private nursing home in Edinburgh, and for two years staff nurse in Longmore Hospital, Edinburgh.

Exeter Infectious Diseases Hospital.—Miss Ellen Ogden has been appointed matron. She was trained for four years at Salford Royal Hospital, and has since been nurse under the Metropolitan Asylum Board; charge nurse of diphtheria wards, Borough Hospital, Croydon; matron, Borough Hospital, West Bromwich, and nurse at the Children's Hospital, Great Ormond Street, London.

### Minor Appointments.

Scarborough Hospital and Dispensary.—Miss Dora Clark has been appointed charge day nurse. She was trained at Norfolk and Norwich Hospital for three years.

Derby Royal Infirmary.—Miss Kate Harrison has been appointed ward sister. She was trained at the General Hospital, Nottingham, where she has since been out-patient nurse.

### To Nurses.

We invite contributions from any of our readers, and shall be glad to pay for "Notes on News from the Nursing World," or for articles describing nursing experiences, or dealing with any nursing question from an original point of view. The minimum payment for contributions is 5s., but we welcome interesting contributions of a column, or a page, in length. It may be added that notices of entertainments, presentations, and deaths are not paid for, but, of course, we are always glad to receive them. All rejected manuscripts are returned in due course, and all payments for manuscripts used are made as early as possible at the beginning of each quarter.

### The National Union of Women Workers at Brighton.

THE Annual Conference of the National Union of Women Workers was held last week at Brighton. The town was full of well-known faces; the Hon. Mrs. A. T. Lyttelton, wife of the Bishop of Southampton (President of the Union), was, of course, there, and amongst others who took a prominent part in the proceedings were the Countess of Aberdeen, Lady Louise Loder, Lady Laura Ridding, Lady Battersea, Mrs. Henry Fawcett, Mrs. Humphry Ward, Mrs. Burgwin, Miss Flora and Miss Louisa Stevenson, Mrs. H. J. Tennant, Miss Alice Busk, Mrs. Bryant, D.Sc., Mrs. Flora Annie Steel, Mrs. Creighton, and Mrs. Bishop, F.R.C.S.

Mrs. Humphry Ward's paper on the "Training of Afflicted Children," in which she gave an account of what is being done at the special school in Tavistock Place on behalf of invalid and crippled children, was full of interest and suggestions for a further extension of this good work of which Mrs. Burgwin, Superintendent of the L. S. B. Special Schools, is a warm advocate. The treatment of "Neurotic Children" was dealt with, at a special meeting, by Mrs. Dickinson Berry, M.D., Dr. Helen Boyle, and Miss Ellice Hopkins. The subject of "Inebriate Homes," the need for which becomes more urgent every day, was the subject of a paper by Dr. Branthwaite, of the Home Office, and temperance and educational matters also formed part of the programme.

### Hospital Boards.

Coming to the subject chiefly interesting to hospital workers, the "Work of Women on Hospital and other Boards," a paper was contributed by Miss Louisa Stevenson (Governor Edinburgh Infirmary), and another by Miss Georgiana Hill, both of which gave scope for a good deal of discussion. Miss Stevenson called attention to some of the matters which especially claim consideration from women members of hospital boards, such as the quality and cooking of the food, nurses' hours of work, and other details of management. She deprecated the substitution of ladies' committees for the admission of women on the governing body, combating the idea that women are only needed in the interests of the patients of their own sex, and of children. They were wanted, she contended, for the sake of men as well. Miss Stevenson took the opportunity of extolling a system of state registration for nurses as a cure for the "present unsatisfactory state of things."

Miss Georgiana Hill's paper was mainly concerned with showing how very rarely women are now admitted on hospital boards, and the curiously strong objection to the presence of women on the managing body which has been manifested by various institutions, such, for example, as the Hospital for Incurables, at Putney, where Miss Hill has been endeavouring to secure their election on the board. Of the larger London general hospitals, Miss Hill showed that none of those with medical schools, St. Bartholomew's, Guy's, St. Thomas's, St. George's, St. Mary's, Westminster, Charing Cross, the London, have either mixed boards or ladies' committees; the Royal Free Hospital is an exception, but this is by special arrangement with the London School of Medicine for Women. In cases, such as the Edinburgh Royal Infirmary, where women have seats on the board, the arrangement has been found to work well, and Miss Hill insisted "there is no reason why men should arrogate to themselves the entire control in a hospital board any more than the entire control in a school board or board of guardians." Mrs. W. J. Evans emphasised a point made by Miss Stevenson with regard to the need for care in selecting women to serve on hospital boards. It was essential that they should possess proper qualifications for such a position.

### English Women in India.

Mrs. Flora Annie Steel read a striking paper on "The Lives of English Women in India." She was often asked, she said, if the literary presentment of the lives of English women given in Mr. Kipling's Indian stories, and in her own, was true to life. The admission that such a presentment was largely true did not really carry a sweeping condemnation of Anglo-Indian women. In India the society life of English women was not leavened, as it was in England, by useful, charitable, and philanthropic work. Indeed, to a great extent, it was not leavened even by family life. Outside the ranks of missionary effort there was little or no kindly, charitable, or neighbourly work done by English ladies. There were two reasons for this strange aloofness. One was that not one in a thousand of the English women who went to India took the trouble to learn the language of the country. Another reason was the disinclination of most Englishmen to allow their womenkind to mix themselves up in any way with native society; and this was by far the most serious obstacle to change in the future. There was no country in the world in which the influence of women was so much deprecated by those in authority as it was in India. It was a thesis of hers that English women were really themselves responsible for this gulf between the rulers and the ruled, and it was her hope that they would yet undo the evil they had done.

### The Housing of the Poor and other Problems.

A couple of very able papers were also read at the evening meeting on Wednesday, under the presidency of Mrs. Henry Fawcett, by Mrs. J. R. Macdonald and Miss Jane Escombe, on "the Housing Problem"; and on Thursday Mrs. Percy Bunting tackled the almost equally difficult problem of "Training for the Proficiency of Domestic Service"; while Mrs. Aldrich dealt with that of "The Management of the Modern Household." The discussion on the former paper was very spirited, and was followed later in the day by excellent addresses from Lady Battersea, and Mrs. Creighton, and Lady Hammick who treated respectively the topics of "Temperance," "A Purpose in Life and Literary Culture," and "Home Life."

### The Training of Poor Law Officials.

In the course of an interesting paper on "The Training and Supply of Poor Law Officials," Mrs. Healey, of Oxford, said it was generally recognised now that for nursing the sick some training was essential, and the system of three years' training had been generally adopted. "Their contention was that in the Workhouse, not only for the nursing of the body, but for the controlling of the wills, habits, and tempers of the inmates, men and women were wanted who were prepared by training. Both Master and Matron had most complicated and onerous duties. . . . . But what preparation had those Masters and Matrons? Sometimes they had shown special capacity in subordinate positions; sometimes chance settled the appointment. Next to the heads of the big, sad world of which they were thinking, she thought the training of laundry matrons and those who were giving attendance to epileptics, the insane, and little children, was most important. No one unfamilar with Workhouse difficulties could imagine the task of laundry matrons. The most heedless and vicious women were put into the laundry—at least that had been her experience—as there they could have most supervision and also because there was scope for unskilled labour. To deal with those women was a task of indescribable difficulty. They wanted in that position a woman who knew her trade and who could enforce obedience, and one who yet had the bowels of mercy."

Mrs. Healey concluded her paper, amid much applause, by expressing her conviction that there was no reason why gentlewomen should not train for posts in workhouses.

EPILEPTICS AND THE FEEBLE-MINDED.

Mrs. Mercer dealt with "The Local Government proposals for dealing with the Epileptic and Feeble-minded." In reviewing the chief recommendations of the circular letter of August 4th to the Boards of Guardians, which has reference to the principle of separating, the means of providing separate institutions, and the cost of maintenance, she said there was a class of institution altogether ignored by the Local Government Board, viz., those founded and maintained by voluntary effort. Voluntary effort should be encouraged, and there should be direct provision for bringing it within the scope of the system. On the whole, the main proposals claimed sympathetic attention, and could be carried into law if the Chancellor of the Exchequer could see his way clear to the financial side of the scheme. After giving a *résumé* of the system followed in the colony of Craig, in the Genesee Valley, and the excellent results there obtained, Mrs. Mercer ended by earnestly appealing to guardians throughout the land to be stirred to increased activity, remembering that "success could only come through the sustained and persistent efforts of individuals."

On Friday evening a reception by the Mayor and Mayoress of Brighton was given at the Pavilion, and on Saturday there was a special service at the parish church, with a sermon by the Bishop of Chichester.

### Everybody's Opinion.

[Correspondence on all subjects is invited, but we cannot in any way be responsible for the opinions expressed by our correspondents. No communication can be entertained if the name and address of the correspondent is not given, as a guarantee of good faith but not necessarily for publication, or unless one side of the paper only is written on.]

"A POSSIBLE DANGER."

"ONE WHO KNOWS" writes: I should like to reassure the Matron of one of the London Hospitals, "who is rather afraid that the nurses who return to ordinary ward work after their exciting experiences at the Cape, &c., will find their work tame, and grow restless." She will find, I am convinced, rather, that they will be improved in every way. They will be, as she herself half tremblingly hopes, "more resourceful in emergency," more gentle and tender, more imbued with the spirit of a real true womanly nurse. Has not the South African Sister tended a husband for one woman, nursed the "only son" of another, soothed the delirium of many a brave, bright youth who had "left his girl behind him?" She has seen, and heard, and lived through some of the saddest things that a woman can bear on God's earth. "Matron" need not fear that she will return home only craving for excitement. Excited, and perhaps for a few short weeks restless, she may possibly feel; but the work she has done, the remembrance of the way in which all in this terrible war, "Tommies," officers, wives, children, and prisoners have borne their hardships; the recollections of the endurance, and the unselfishness will make of her a higher, nobler, better woman. Did Florence Nightingale's work unsettle her? or make her crave for excitement? Has not her experience rather made her such a woman as all the nursing world honours, looks up to, and fain would copy? Now is your time, nurses! I call on you all, who are returning to your quieter duties, to uphold me in what I have said; to prove that my little defence is true; show "Matron" and all the British public that a British nurse is like a British soldier, not only ready and willing for "active service," fearless and dauntless in the face of danger and difficulty (*that* no one seems to doubt!) but also equally ready to "serve in the quiet ways."

VISITING NURSES FOR THE MIDDLE CLASSES.

"H. B." writes: I am much interested in this subject, as I hope during the winter to do a deal of visiting. While engaged in private work during the last few years I have seen the great need of nurses who attend ladies in poor circumstances for an hour or two daily at the rate of—say—10s. 6d. or 12s. weekly. I, too, think 3s. 6d. per hour a large sum for anyone to pay who earns at most from £2 2s. to £3 3s. weekly. If nurses would make a sliding scale, and charge higher fees where it can be well afforded, they would benefit those for whom daily nursing is a necessity, and whose condition, when ill, is often wretched and miserable in the extreme, through lack of means to pay a nurse's full fee.

"AN OLD ST. THOMAS'S NURSE" writes: I am very glad to see that the middle classes are having a thought given to them at last. It has often puzzled me why the really needy middle-class ladies and gentlemen are not cared for when ill by the Queen's Jubilee Nurses. Where can be found greater distress than among the poor but refined people who struggle on and often are obliged to deny themselves bare necessaries of life to pay their way and keep up a respectable appearance, which they must do to get employment at all? Very few know the untold misery and privation borne silently and patiently by many, and to such what an inestimable boon would be a gift of free nursing! I hope some day we shall see this great want supplied. Until then, I am afraid, however small a fee the visiting nurse charges these patients, it will be a great strain upon them; though I am quite sure that the visiting nurse will be very lenient in such cases. For ordinary middle-class nursing a fee of 3s. 6d. an hour, or part of an hour, should be the lowest recognised fee. The nurse will soon be able to judge if the patient can afford to pay her 3s. 6d. a visit. I have more than once reduced my fee, without being asked, when I have seen and known the people I was nursing could not afford to pay two guineas per week. No doubt many other nurses have done the same. It may be worth while to compare the life of an average private resident nurse's with that of the visiting nurse. The resident nurse attends her patient under very different circumstances. For instance, the resident nurse has her patient on the spot; no rush for 'bus or train. She has her comfortable surroundings, and her work is not heavy hour after hour, unless in very exceptional cases, when she usually has the help of another nurse. Her meals are prepared, and served without any thought or trouble to her, and she is usually surrounded with every comfort in moderation while attending her patient; her fee, meals, room, and all that she is provided with, therefore, is quite equivalent to three and a half guineas. The visiting nurse, on the other hand, must pay for everything she has—her food, rooms, attendance, &c., which is fairly considerable. And it would be well if the public would take all these things into consideration before they call a fee of 3s. 6d. prohibitive or preposterous. There is also the great discomfort which the visiting nurse must face in getting to her patients. She must start early in the morning, and may have some distance to go, as all her patients cannot be quite at hand; and this she must do in all weathers, pouring rain, snow, fog, or blazing sunshine, exposed in winter to heated rooms, and the sudden coming out into the cold again. No one can understand the strain on a nurse's health and strength as she attends each patient hour after hour.

I am quite certain that the visiting nurse will be the greatest comfort to those in lodgings, chambers, or any house where the resident nurse is not desirable, and I do not think that there will be much difficulty about the fee. For the benefit of those who contemplate taking up this line of life, and intend nursing the middle classes or others by the hourly visit, I should like to say a few words. Think well before you give up regular resident engagements, or the connection with institutions or doctors; you will not find it easy to resume it. Although this life has its advantages, yet it has some disadvantages, and only those who are really strong should attempt it. Let me beg of those who do take it up to make time for their food as regularly as they possibly can, and have someone responsible for their home. Don't try and combine home duties with professional engagements; but when you go home tired, rest. Be with some kind landlady who will see you have a nice hot meal and a comfortable fire when you return. Unless you give reasonable attention to these matters you will slowly but surely break down, as so many in our profession have done. I have been a private nurse for several years, I have also been a visiting nurse and masseuse, so that I believe I know all the advantages and disadvantages of visiting nursing.

### For Reading to the Sick.

ALL SAINTS DAY.

"I BELIEVE in the communion of saints."

"I would not have you to be ignorant, brethren, concerning them which are asleep, that ye sorrow not, even as others which have no hope."—1 Thess. iv. 13.

Lord make me one with Thine own faithful ones,
Thy saints who love Thee and are loved by Thee :
Till the day break and till the shadows flee,
At one with them in alms and orisons ;
At one with him who toils and him who runs
And him who yearns for union yet to be ;
At one with all who throng the crystal sea
And wait the setting of our moons and suns.
Ah, my beloved ones gone on before,
Who looked not back with hand upon the plough!
If beautiful to me while still in sight
How beautiful must be your aspects now ;
Your unknown, well-known aspects in that light
Which clouds shall never cloud for evermore.
*Christina Rossetti.*

Let us learn from this communion of saints to live in hope. Those who are now at rest were once like ourselves. They were once weak, faulty, sinful; they had their burdens and hindrances, their slumbering and weariness, their failures and their falls. But now they have overcome. Their life was once homely and commonplace. Their day run out as ours. Morning and noon and night came and went to them as to us. Their life, too, was as lonely and sad as yours. Little fretful circumstances and frequent disturbing changes wasted away their hours as yours. There is nothing in your life that was not in theirs; there was nothing in theirs but may be also in your own. They have overcome, each one, and one by one; each in his turn, when the day came, and God called him to the trial. And so shall you likewise.—*H. E. Manning.*

Let us realise also that we can never be lonely or forsaken in this life. Shall they forget us because they are "made perfect?" Shall they love us the less because they now have power to love us more? If we forget them not, shall they not remember us with God? No trial, then, can isolate us, no sorrow can cut us off from the Communion of Saints. Kneel down, and you are with them, lift up your eyes, and the heavenly world, high above all perturbation, hangs serenely overhead; only a thin veil, it may be, floats between. All whom we loved, and all who loved us, whom we still love no less, while they love us yet more, are ever near, because ever in His presence in whom we live and dwell.—*H. E. Manning.*

We are compassed about by a cloud of witnesses, whose hearts throb in sympathy with every effort and struggle, and who thrill with joy at every success. How should this thought check and rebuke every worldly feeling and unworthy purpose, and enshrine us, in the midst of a forgetful and unspiritual world, with an atmosphere of heavenly peace! They have overcome—have risen—are crowned, glorified; but still they remain to us, our assistants, our comforters, and in every hour of darkness their voice speaks to us: "So we grieved, so we struggled, so we fainted, so we doubted; but we have overcome, we have obtained, we have seen, we have found—and in our victory behold the certainty of thy own."—*H. B. Stowe.*

We by enemies distressed,
They in Paradise at rest ;
We the captives, they the freed,
We and they are one indeed,
One in all we seek or shun,
One, because our Lord is one,
One in heart and one in love,
We below and they above.—*J. M. Neale.*

### Notes and Queries.

**The Editor is always willing to answer in this column, without any fee, all reasonable questions, as soon as possible.**

**But the following rules must be carefully observed :—**

1. **Every communication must be accompanied by the name and address of the writer.**
2. **The question must always bear upon nursing, directly or indirectly.**

**If an answer is required by letter a fee of half-a-crown must be enclosed with the note containing the inquiry.**

*Not Responsible.*

(49) Can you tell me of a home where a man of 33, who has been a confirmed drunkard, and who is now not responsible for his actions, could be placed? His people would contrive to pay a small sum weekly.—*Miserere.*

How would the Royal Victoria Home, Horfield, Bristol, suit? Only working patients are received. Terms, 10*s*. a week. Apply the Warden. But if a man is not responsible for his actions surely he is of unsound mind, and if of unsound mind he cannot be received for profit, except under certificate as a lunatic.

*Maternity Fees.*

(50). I should be thankful if some one would tell me about maternity fees. Say I am kept waiting a fortnight, am I expected to give the time?—*Anxious.*

Certainly not. It is usual to charge half fees during the period spent waiting. In future, have your charges for the confinement, and for any time you may be kept waiting, printed on your professional card, and see that each patient has one before the engagement is settled. This leaves no room for dispute.

*Return Passage.*

(51) I am anxious to return to Cape Town, where I was engaged as monthly nurse some four years ago. I am a good sailor, and would like to take charge of a patient on the out-going voyage. Could you tell me how best to advertise?—*Nurse Emma K.*

You would receive most help from a regular advertising agent, who would send you a list of papers and prices.

*Nursing Appointment.*

(52) Can you give me the address of nursing institutions either in Ceylon or South Africa, and tell me how to make application?—*M. L. M.*

The Ceylon Nursing Association, or the Victoria Nurses' Institute, Cape Town, Cape Colony. Apply to the Lady Superintendents.

"*Melancholic.*"

(53) Could you kindly tell me of a home where a poor person, who is very melancholic, could be received for a small sum?—*Nurse Barbara.*

You might hear of a suitable cottage home through the "After-care Association for Persons Dismissed from Asylums," Church House, Dean's Yard, Westminster, S.W.

*St. George's Infirmary.*

(54) 1. Is the training at St. George's Infirmary, Fulham Road, good? Would it qualify a nurse holding a three years' certificate for a good post? 2. Can you give me the names of a few hospitals paying salary from the first, in or near London?—*E. S.*

It is an important infirmary, and grants the usual certificates. 2. See the "Nursing Profession: How and Where to Train." We have not space to give lists of institutions.

*Army Nursing Reserve.*

(55) Would you kindly tell me to whom I should apply in order to get on the Army Nursing Reserve?—*Enquirer.*

Apply, the Hon. Secretary, 18 Victoria Street, Westminster, S.W.

*Massage.*

(56) Can I get free training in massage at any of the nerve hospitals in return for a term of service? I have had only three months' training as nurse, but I have had three years' experience in nursing.—*L. C. S.*

No; but you can obtain a course of instruction in massage at a moderate fee at the National Hospital for the Paralysed and Epileptic, Queen's Square, Bloomsbury, W.C.

*Tropical Diseases.*

(57) Where is there a hospital for training in tropical fevers and diseases? 2. Can a certificated nurse enter in any other capacity than as probationer? 3. Where is there a leprosy hospital, and what qualifications are necessary to become a Sister in one?—*H. S.*

Schools have been founded for the study of tropical diseases at Greenwich and Liverpool. 2. At the former trained nurses are received for a course of special instruction. 3. See "Burdett's Hospitals and Charities" for list of leper hospitals. Particulars as to qualification must be sought from the authorities at the different institutions.

*A Trained Nurse.*

(58) What constitutes a trained nurse? Will a three years' certificate of efficiency from a general hospital of 40 beds after courses of lectures from the visiting physicians and surgeons qualify its holder as a "trained nurse"?—*Perplexed.*

Only training in schools maintaining a resident medical officer is recognised by the Local Government Board as qualifying a nurse for the position of superintendent nurse. However well trained the holder of the above certificate may be, she would not be eligible for that position.

**Standard Books of Reference.**

"The Nursing Profession: How and Where to Train." 2s. net; post free, 2s. 4d.
"The Nurses' Dictionary of Medical Terms." 2s.
"Burdett's Series of Nursing Text-Books." 1s. each.
"A Handbook for Nurses." (Illustrated.) 5s.
"Nursing: Its Theory and Practice." New Edition. 3s. 6d.
"Helps in Sickness and to Health." Fifteenth Thousand. 5s.
"The Physiological Feeding of Infants." 1s.
"The Physiological Nursery Chart." 1s.; post free, 1s. 3d.

All these are published by THE SCIENTIFIC PRESS, Ltd., and may be obtained through any bookseller or direct from the publishers, 28 and 29 Southampton Street, London, W.C.

